# Case Report: Overlapping Syndrome of Anti-NMDAR Encephalitis and MOG Inflammatory Demyelinating Disease in a Patient With Human Herpesviruses 7 Infection

**DOI:** 10.3389/fimmu.2022.799454

**Published:** 2022-04-22

**Authors:** Sisi Li, Minjin Wang, Hancong Li, Jierui Wang, Qi Zhang, Dong Zhou, Jinmei Li

**Affiliations:** ^1^ Department of Neurology, West China Hospital, Sichuan University, Chengdu, China; ^2^ Department of Laboratory Medicine, West China Hospital of Sichuan University, Chengdu, China; ^3^ West China School of Medicine, West China Hospital of Sichuan University, Chengdu, China

**Keywords:** anti-NMDAR encephalitis, optic neuritis, HHV-7 infections, case report, MOG antibodies and NMDAR encephalitis overlapping syndrome (MNOS)

## Abstract

**Objectives:**

This study reported a case of overlapping anti-N-methyl-D-aspartate receptor (NMDAR) encephalitis and myelin oligodendrocyte glycoprotein (MOG) inflammatory demyelinating disease with human herpesviruses 7 (HHV-7) infection.

**Methods:**

The detailed clinical characteristics, neuroimaging features, and outcomes of the patient were collected. Polymerase chain reaction (PCR), cell-based assay (CBA) and the tissue-based indirect immunofluorescence assay (TBA) were used for diagnosis.

**Results:**

The clinical manifestations included headache, dizziness, fever, optic neuritis, and epileptic-seizures. Brain magnetic resonance imaging (MRI) showed hyperintensities involving the left frontal, orbital gyrus and bilateral optic nerve with substantial contrast enhancement. Moreover, test for HHV-7 DNA by using the next generation sequencing metagenomics and polymerase chain reaction showed positive result in CSF but not in the serum samples. Anti-HHV-7 IgM and IgG antibodies were detected in both the serum and cerebrospinal fluid. NMDAR antibodies (1:10) were found positive in the patient’s CSF by a cell-based assay, and MOG antibodies were positive in the serum (1:10) and CSF (1:32). The patient appeared to respond well to immune therapy and it was found that the clinical symptoms including epileptic-seizure as well as headache were relieved and cerebral lesions almost disappeared after the treatment. However, his vision was not completely restored even at the 8-month follow-up, especially the vision in his right eye which was more seriously damaged.

**Discussion:**

We report a rare case of MOG antibodies and anti-NMDAR encephalitis overlapping syndrome (MNOS) with HHV-7 infection for the first time. The possibility of MNOS needs be considered when optic neuritis occurs in the patients diagnosed with anti-NMDAR encephalitis. Besides, immunotherapy should be initiated as early as possible to improve the treatment outcomes and facilitate complete cure.

## Background

Anti-N-methyl-D-aspartate receptor (NMDAR) encephalitis is an immune-mediated disorder that is associated with IgG antibodies to the GluN1 subunit of the NMDA receptor (1, 2). The clinical manifestations of patients with anti-NMDAR encephalitis include psychosis, memory deficits, seizures, language disintegration, abnormal movements, and autonomic as well as breathing instability (3). Recently, it has been observed that an underlying tumor (usually teratomas) (4) and virus infections (5) can serve as the two triggers in the development of anti-NMDAR encephalitis. Herpes simplex virus (HSV) -1 acts as one of the most commonly reported viral triggers for anti-NMDAR encephalitis (6, 7), which has been detected in 27% of patients with HSV encephalitis (8). For the pathophysiological mechanisms underlying viruses-induced encephalitis, it had been proposed that virus-mediated brain tissue damage could potentially lead to exposure of the normally sequestered neuronal cell antigens or that the cause of autoantibody production could be possible “molecular mimicry” of viral proteins due to the striking similarity between NMDAR and viral antigenic peptides or antibodies (8).

Several cases have recently reported the coexistence of anti-NMDAR and myelin oligodendrocyte glycoprotein (MOG) antibodies (9–12). For instance, a study has reported that 11.9% of the patients with MOG antibody-associated inflammatory demyelinating disease had anti-NMDAR encephalitis, which had been defined as MOG antibody disease and anti-NMDAR encephalitis overlapping syndrome (MNOS) (10). Among 184 patients with anti-NMDAR encephalitis, 2.7% patients were identified as having overlapping MOG antibody disease (12). The clinical manifestations included headache, fever, seizures, cognitive impairment, psychiatric disorders, disturbance of consciousness, and the symptoms of demyelination (12).

Human Herpesviruses 7 (HHV-7) is a β-herpesvirus, which usually infects during the childhood and can thereafter exist in a lifelong latent state with possible reactivation in case of immunodeficiency (13, 14). The virus exhibit multiple immunomodulatory functions by encoding some specific viral proteins, which could effectively facilitate evasion of virus-The virus can exhibit multiple immunomodulatory functions by encoding some specific viral proteins, which could effectively facilitate evasion of virus specific immune response and can modify the host microenvironment to promote the viral persistence (15). When investigating HHV-7 CNS disease, the primary infection can be diagnosed through combining the CSF polymerase chain reaction (PCR) with serology (16). To our best knowledge, such case of HHV-7 infection and MNOS has not been reported previously in the literature. Here, we describe the case of a patient with overlapping MOG antibody disease and anti-NMDAR encephalitis who was also found to have HHV-7 infection.

## Case Report

**Figure 1:**
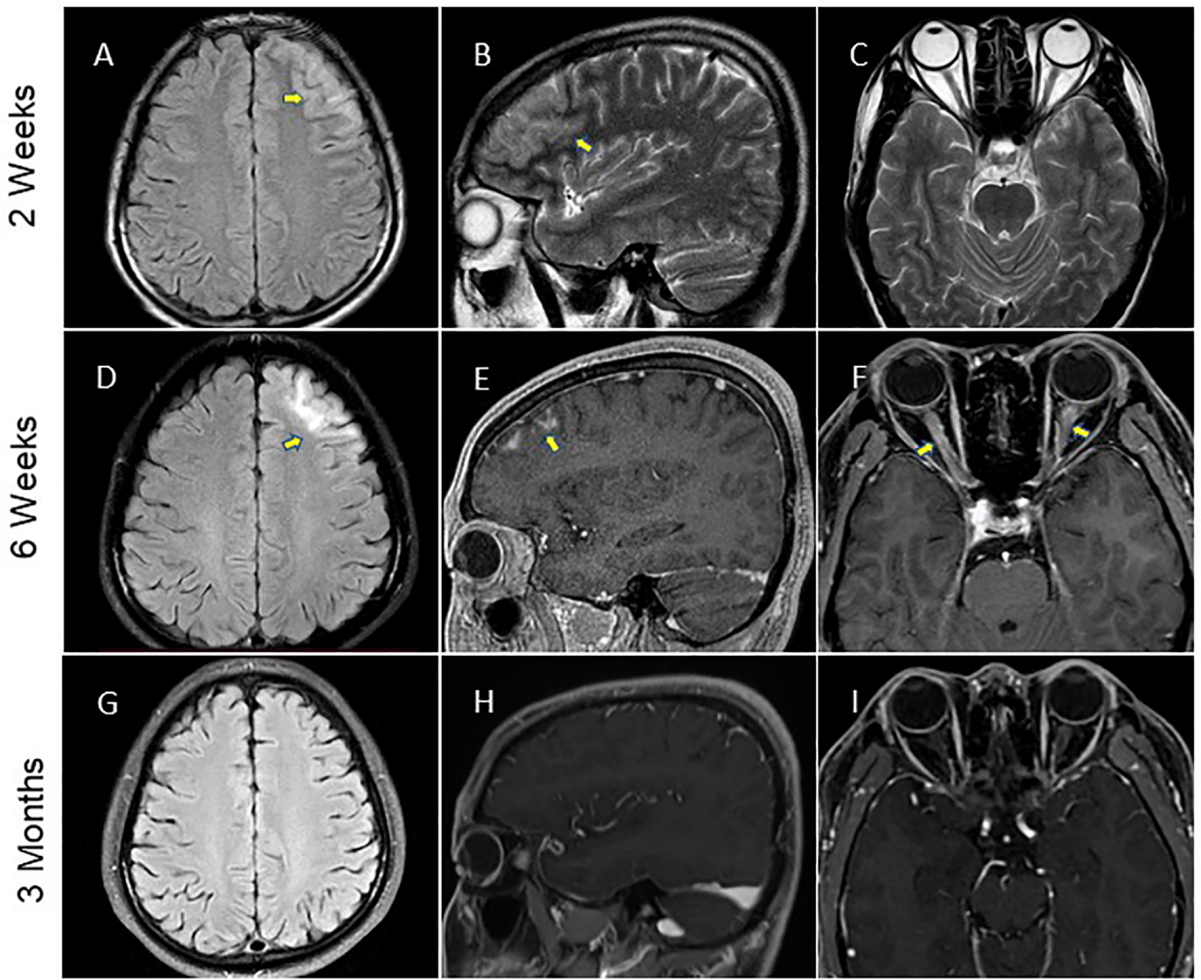
Brain MRI performance. **(A–C)** The MRI data of patient at two weeks after the symptom onset showed hyperintensity of the left frontal lobe on fluid-atten uated inversion recovery (FLAIR) imaging and T2-weighted imaging (arrows). **(D–F)** MRI of the brain was repeated at 6 weeks and showed enhancement of the left frontal lobe lesion involved the bilateral optic nerve (arrows) on FLAIR imaging conducted with gadolinium contrast. **(G–I)** Brain MRI scan performed at three months after initial symptom onset depicted a significant improvement of the imaging abnormality.

A 28-year-old man was brought to a local hospital in April 2021 because of the secondarily generalized epileptic-seizure, which presented itself with a sudden loss of consciousness, staring eyes, and stiffness of the limbs and convulsions. The various symptoms lasted for several minutes and terminated before reaching the hospital, and the patient was unable to recall the onset of this seizure. The patient had only one epileptic episode. The type of seizure was considered as focal impaired awareness with motor onset seizure (17). For the past one week, he suffered from headache and dizziness, but his medical history was unremarkable. Magnetic Resonance Imaging (MRI) of the brain showed significant hyperintensity of the cortical and subcortical of the left frontal lobe (**Figures 1A–C**). Thereafter, lumbar puncture was performed two days later, which revealed an increased intracranial pressure (250mmH2O), high protein levels (1.28 g/L) and increased accumulation of cells (610 white blood cells/mm3), but normal glucose and chloride levels. During hospitalization, the patient occasionally suffered with fever with a temperature of around 38.5°C. He was initially diagnosed with meningoencephalitis. Therefore, he was administered empirical intravenous (IV) anti-viral (acyclovir, 500mg/d, Q8h) and antibiotic (ceftriaxone sodium, 2000mg/d) therapy, and the above symptoms were gradually alleviated. After one week, the patient developed blurred vision in the right eye, which deteriorated markedly with binocular vision loss after 10 days and continued to worsen continuously over the following month. Repeated brain MRI showed enlarged lesion involving the left frontal, orbital gyrus and bilateral optic nerve with substantial contrast enhancement (**Figures 1D–F**). Hence, he was transferred to our tertiary care center for further diagnosis. Neurological examination revealed binocular vision loss with only light perception, and lateral visual field was impaired (**Supplementary Figures 2A, B**). Muscle weakness, sensory nervous system, cerebellar functions, Babinski sign and meningeal irritation signs were observed to be in the normal range.

**Figure 2:**
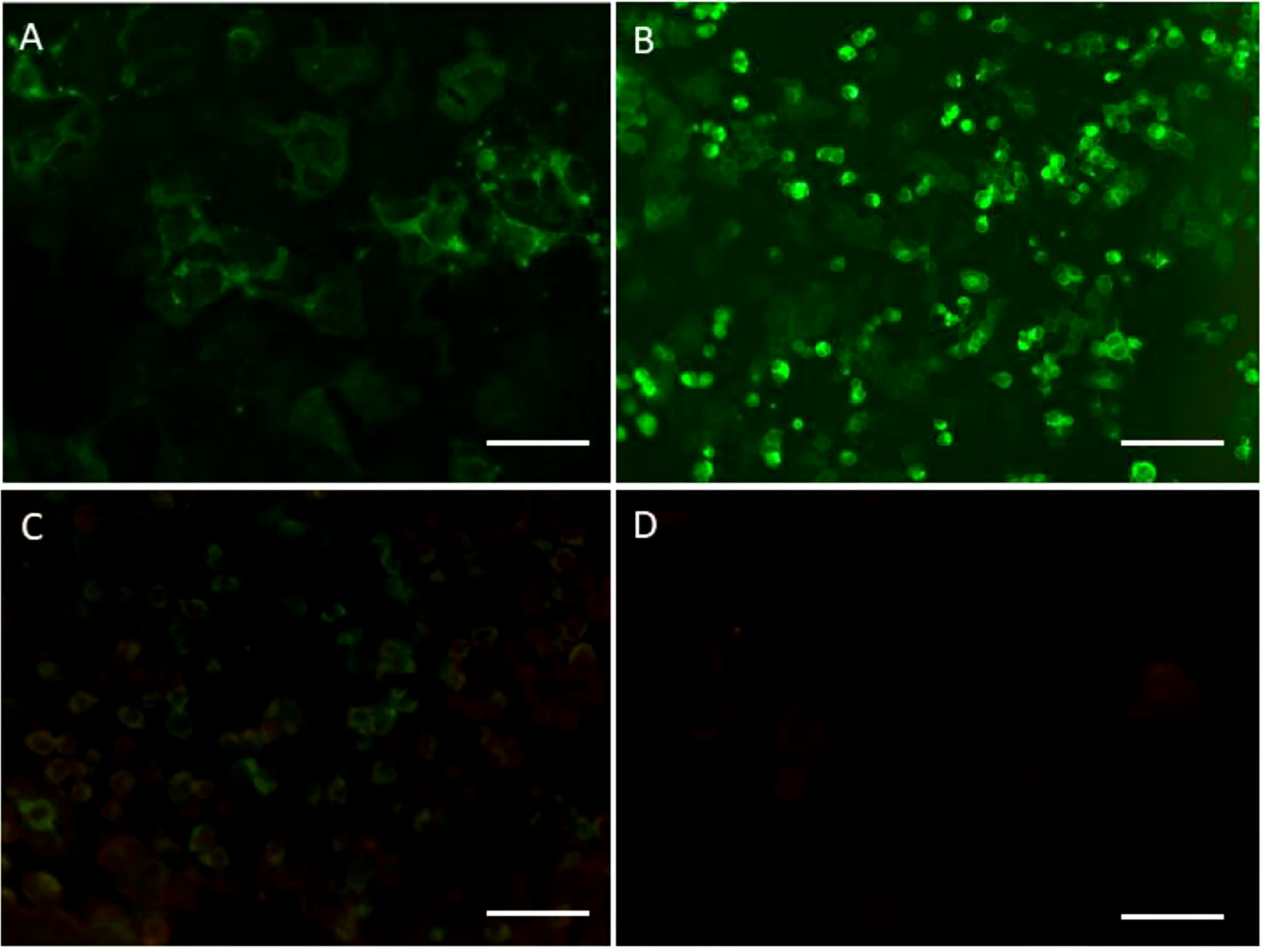
**(A)** Cerebrospinal fluid (CSF) showing the binding to the surface of the cells expressing anti-NMDAR receptors (NMDAR) (1:10) (scale bar 20μm). **(B)** Myelin oligodendrocyte glycoprotein (MOG) antibodies (1:32) were detected in the CSF (scale bar 100μm). **(C)** MOG antibodies (1:1) were detected in serum (scale bar 100mm). **(D)** GFAP antibodies were found negative in the CSF (scale bar 100mm).

Electroencephalograph (EEG) revealed a small amount of scattered slow waves. Repeated lumbar puncture depicted lower white blood cell count (11 cells/mm3) and protein (0.51 g/L) level. Anti-HHV-7 IgM and IgG antibodies were detected in both the serum and cerebrospinal fluid (CSF). CSF testing for HHV-7 RNA by next generation sequencing (NGS) metagenomics and polymerase chain reaction (PCR) yielded positive result. All other CSF tests for the neurotropic viruses and other bacteria displayed negative results. Upon search for anti-neural antibodies (to anti-NMDAR, anti-LGI1, anti-CASPR2, anti-AMPAR, anti-GABABR, anti-DPPX, and anti-mGLuR5), NMDAR antibodies (1:10) were found positive in the patient’s CSF by cell-based assay (CBA) (Euroimmun, Lübeck, Germany) (**Figure 2A**). We also used CBA (Euroimmun, Lübeck, Germany) to detect CNS demyelinating antibodies (AQP4, MOG, GFAP), MOG antibodies were found positive in CSF (1:32) (**Figure 2B**) and the serum (1:10) (**Figure 2C**), whereas both AQP4 antibody (not shown) and GFAP antibody (**Figure 2D**) were negative. Besides, we found a weak fluorescence response in the TBA detection, which indicated that the disease was immunological in nature (**Supplementary Figures 1A, B**). The various tests conducted for the paraneoplastic antibodies, rheumatological autoantibodies, blood routine examination, biochemistry, tumor markers, thyroid profile, mycobacteria, treponema pallidum, human cytomegalovirus, and viral serological were all found to be negative (**Supplementary Table 1**). NGS metagenomics tests, TBA tests, and CBA tests for anti-neural antibodies and CNS demyelinating antibodies also were performed at the Center for Precision Medicine, West China Hospital, Sichuan University. Study methods are provided in the **Supplementary Material**.

## Treatment and Outcomes

The patient was prescribed IV ganciclovir for 21 days and IV methylprednisolone (IVMP) for 5 days (1000 mg/day). He was continued with immunosuppressive treatment with prednisolone (1mg/kg/d) after discharge.

Two months after the discharge, it was found that the patient was free of headache and seizures. Repeated brain MRI scan results showed that the lesions in the left frontal cortex as well as the sub-cortex had almost disappeared, and bilateral optic nerve enhancement was also significantly diminished (**Figures 1G–I**). However, the patient’s vision was not completely resolved. His visual acuity was 20/167 in the right eye, and 20/33 in the left eye, and his field of vision was still impaired (**Supplementary Figures 2C, D**). As a result of the long-term hormone therapy, the patient gained weight and developed acne on the skin of his face. Blood routine examination and liver as well as kidney function showed no obvious abnormality. The patient started the second-line immunosuppressant therapy with mycophenolate in August 2021.

**Figure 3:**
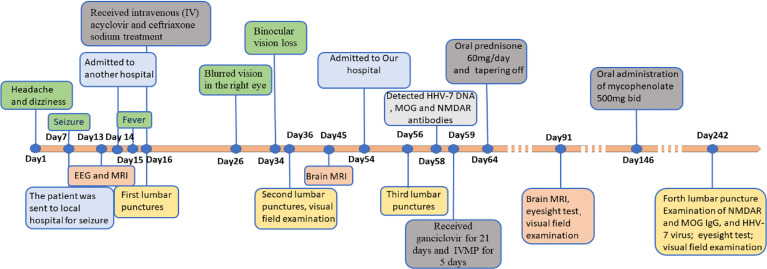
Timeline with clinical manifestation, treatment progression and diagnosis time. EEG, Electroencephalograph; MRI, Magnetic Resonance Imaging; IVMP, Intravenous methylprednisolone; HHV-7, Human Herpesviruses-7; NMDAR, Anti-N-methyl-D-aspartate receptor; MOG, Myelin oligodendrocyte glycoprotein.

After being treated with the second-line immune therapy, the patient no longer suffered from the recurrent headache and seizure. His visual acuity was found to be 20/80 in the right eye, and 20/25 in the left eye, and his field of vision was marginally better than before (**Supplementary Figures 2E, F**). A follow-up performed 6 months after the discharge, the patient was found negative for anti-HHV-7 IgM antibodies, anti-HHV-7 IgG antibodies, HHV-7 DNA, MOG antibodies (**Supplementary Figure 1C**), and anti-NMDAR antibodies (**Supplementary Figure 1D**) in the CSF analysis, and weakly positive for anti-HHV-7 IgG antibodies in the serum. Despite remaining partially visually impaired, his daily life was not significantly affected and he had returned to work. The whole disease course and the therapeutic modalities used have been summarized as a graphical abstract (**Figure 3**).

## Diagnostic Assessment

The patient’s clinical manifestations such as fever, headache, and epileptic seizure were similar to the presentation of the HHV-7 encephalitis. The finding of the viral DNA in the CSF by PCR and NGS but not in the serum samples suggested that the primary HHV-7 infection with invasion of the CNS and consequential disease had occurred (17). Besides, anti-HHV-7 IgM and IgG antibodies were observed to be positive in both CSF and serum, thus HHV-7 encephalitis was considered in this patient. Additionally, according to the clinical manifestations of bilateral optic neuritis, imaging features of MRI, positive NMDAR antibodies as well as MOG antibodies in the CSF, and a good response to IVMP therapy, the patient was diagnosed as MNOS. Since the first wave of the symptoms were headache, dizziness, and seizure, which were relieved after antiviral and antibacterial treatment, whereas the second wave of symptoms (optic neuritis) appeared on the 26^th^ day of onset, we believed that HHV-7 encephalitis was secondary to MNOS. At last, a final diagnosis of overlapping syndrome of anti-NMDAR encephalitis and MOG inflammatory demyelinating disease with human herpesviruses 7 infection was made.

## Discussion

We report for the first time a rare case about a patient who was diagnosed with overlapping anti-NMDAR encephalitis and MOG inflammatory demyelinating disease after HHV-7 infection. Neurotropic viruses linked to anti-NMDAR encephalitis have been previously described in the literature. Several studies have proposed that HSV infections might have meaningful association with the development of anti-NMDAR encephalitis (18, 19). Except for HSV, recent clinical observations have postulated that other viruses may also potentially elicit anti-NMDAR encephalitis. Varicella zoster viruses (VZV) have been detected in the patients with anti-NMDAR encephalitis (20–22), thus acting as a trigger for the disease. In the post-solid organ transplant patients, Epstein-Barr Virus (EBV) and NMDAR antibodies have been reported as double positive in rare infected cases (23). Human parvovirus B19 infection has been reported to be associated with GABAA receptor encephalitis (24). Recently, a case of encephalitis associated to immunoreactivity related to SARS-CoV-2 infection has also been reported (25). Except for case reports, a study based on Mayo clinic neuroimmunology laboratory was conducted to report the frequency of coexisting herpes viruses (HSV-1, HSV-2, VZV, EBV, cytomegalovirus, or HHV-6) and autoantibodies in patients with encephalitis (26). They confirmed that para-infectious autoimmunity may occur in the context of EBV and VZV, and demonstrated that a spectrum of co-existence of herpes viruses and antibodies extends beyond HSV-1 and NMDAR antibodies (26).

HHV-7 is a T-lymphotropic virus, primarily infecting CD4^+^ and CD8^+^ primary lymphocytes (27), and usually occurs during the childhood (28). The common presenting clinical manifestations of HHV-7 encephalitis include fever, headache, seizure, and altered level of consciousness (16). Neurological complications of HHV-7 are very rare in immunocompetent adults, and most of them have been predominantly reported in HIV-positive patients (29) or transplant recipients (30). In immune-competent patients, HHV-7 infections have been associated with severe neurologic disease including encephalitis and Guillain-Barre syndrome in adolescents (16), poly-myeloradiculopathy (14) and limbic encephalitis (31) in adults. The clinical manifestations of the present patient such as fever, headache, and epileptic seizure were similar to those of the HHV-7 encephalitis cases, which may possibly be associated with activation of HHV-7 virus. Moreover, loss of the visual acuity and papilledema were reported as the initial presentation in an immune-competent adult male patient with HHV-7 DNA detection from CSF (32). As almost all adults were infected with HHV-7 in early childhood, delayed primary infection accompanied with manifestations of serious symptoms are exceptionally rare (33). Moreover, only one case has been reported about a child who was diagnosed with MNOS secondary to the viral encephalitis, but the common neurotropic virus (including HSV, EBV, and enterovirus) was not found in CSF samples by PCR analysis (34). To our best knowledge, no case of CNS HHV-7 infection association with MNOS has been documented in the literature.

Optic neuritis is rare in patients with anti-NMDAR encephalitis, but has been often reported in the patients diagnosed with MNOS (9). Chen et al. reported that 37.9% of patients with MNOS had symptoms of optic neuritis, and 46.7% of them displayed abnormal signals on MRI involving cerebral cortical (12). The typical manifestation of bilateral optic neuritis and abnormal signals of the cerebral cortical on MRI for the present case are the specific characteristic of MNOS. Therefore, we detected MOG antibodies by CBA and obtained a positive result. Since the seizure occurred in the first week after the onset of initial symptoms, it was classified as the first wave of the symptoms. In addition, epileptic seizures have also been reported as one of the common manifestations of HHV-7 encephalitis, so it was thought that the epileptic seizure of this patient may be related to HHV-7. Nevertheless, unilateral cortical MRI encephalitis with epileptic seizure has been reported to be closely associated with MOG-antibodies (35, 36), thus the possibility of seizures caused by MOG antibodies should also be considered.

Although most patients with MNOS respond well to the first-line immunotherapy, recurrence still needs to be considered. Steroids combined with second-line immunotherapy can effectively help to reduce the rates of recurrence (12). In our case, the patient was thereafter treated with IVMP combined with ganciclovir antiviral therapy, and the second-line immunosuppressant therapy treated with mycophenolate. In general, the patient responded well to immunotherapy and displayed improved clinical symptoms as well as radiographic findings during the follow-up. Nevertheless, his sight was not completely recovered, especially in his right eye, which was more severely damaged. The results from the study suggested that a longer immunotherapy treatment and follow-up may be required when optic neuritis occurs in patients with MNOS. Besides, immunotherapy should be initiated as early as possible to improve the treatment outcomes and facilitate complete cure.

## Patient Perspective

When my eyesight suddenly failed in both eyes, my world plunged into darkness and I felt very frightened and helpless. But when I received treatment and my eyesight gradually returned, I began to gain confidence. At present, my daily life is basically not significantly affected, but the vision of my right eye has still not been fully restored to normal, and I feel that it might be difficult to restore it completely.

## Data Availability Statement

The original contributions presented in the study are included in the article/**Supplementary Material**. Further inquiries can be directed to the corresponding author.

## Ethics Statement

The patient in this case report provided written informed consent for publication.

## Author Contributions

SL and MW drafted and revised the manuscript. HL collected data and revised the manuscript. JW and QZ collected and interpreted the data. DZ revised the manuscript for content. JL designed the study and revised the manuscript. All authors contributed to the article and approved the submitted version.

## Funding

This work was supported by grants from National Natural Science Foundation of China (Grant No. 81571272) and from science and technology project of Sichuan Province (Grant No. 2019YFH0145).

## Conflict of Interest

The authors declare that the research was conducted in the absence of any commercial or financial relationships that could be construed as a potential conflict of interest.

## Publisher’s Note

All claims expressed in this article are solely those of the authors and do not necessarily represent those of their affiliated organizations, or those of the publisher, the editors and the reviewers. Any product that may be evaluated in this article, or claim that may be made by its manufacturer, is not guaranteed or endorsed by the publisher.

## Supplementary Material

Supplementary Figure 1 Tissue-based indirect immunofluorescence assay (TBA) detected weak fluorescence responses, with specific fluorescence responses in **(A)** interstitial connective tissue (arrows) from the monkey optic nerve section as well as **(B)** Purkinje cells (arrows) from the monkey cerebellum brain section (scale bar 100μm). Cerebrospinal fluid MOG **(C)** and anti-NMDAR **(D)** antibodies were negative by cell-based assay at 8 months of follow-up (scale bar 100μm).

Supplementary Figure 2 Changes of visual filed in different periods after onset: **(A, B)** one month, **(C, D)** three months, and **(E, F)** eight months.

## Abbreviations

HHV, Human Herpesviruses; NMDAR, anti-N-methyl-D-aspartate receptor; VZV, Varicella-zoster virus; EBV, Epstein-Barr Virus; HSV, Herpes simplex virus; FLAIR, fluid-attenuated inversion recovery; PCR, polymerase chain reaction; CBA, Cell-based assay; TBA, Tissue-based indirect immunofluorescence assay; NGS, Next generation sequencing; CNS, Central nervous system; CSF, Cerebrospinal fluid; MOG, Myelin oligodendrocyte glycoprotein; MOGAD, MOG-IgG associated inflammatory demyelinating disease; MNOS, MOG antibodies and NMDAR encephalitis overlapping syndrome; AQP4, Anti-aquaporin 4; GFAP, Glial fibrillary acidic protein; MRI, Magnetic Resonance Imaging; EEG, Electroencephalograph; IV, Injection of vein; IVMP, Intravenous methylprednisolone; IVIG, Intravenous immunoglobulin; NMDA, N-methyl-D-aspartate; anti-GABAB, Anti-gamma-aminobutyric acid-B receptor; LGI1, Leucine-rich glioma-inactivated protein 1; CASPR2, Contactin-associated protein-like 2; AMPAR α-amino-3-hydroxy-5-methyl-4isoxazolepropionic acid receptor; DPPX, Dipeptidyl peptidase-like protein-6; mGluR5 metabotropic glutamate receptor 5.
