# Supplementary figures and images for: Case Report: Overlapping Syndrome of Anti-NMDAR Encephalitis and MOG Inflammatory Demyelinating Disease in a Patient With Human Herpesviruses 7 Infection
Source: Front Immunol. 2022 Apr 22;13:799454. doi: 10.3389/fimmu.2022.799454 (PMC9074690; doi:10.3389/fimmu.2022.799454)

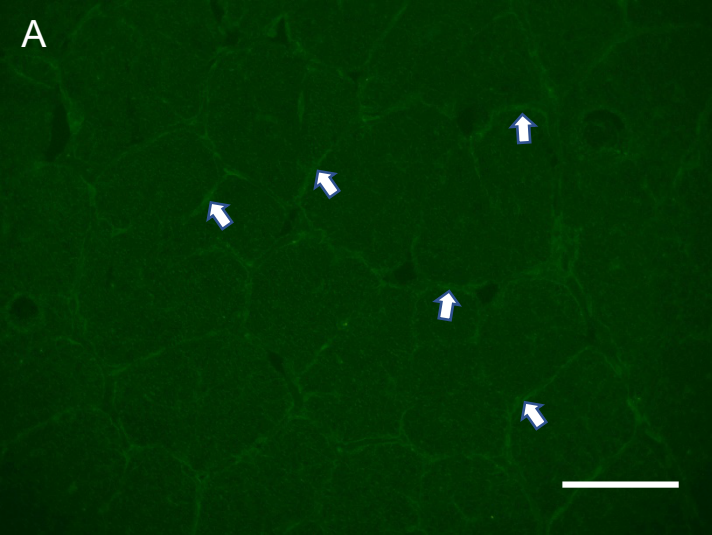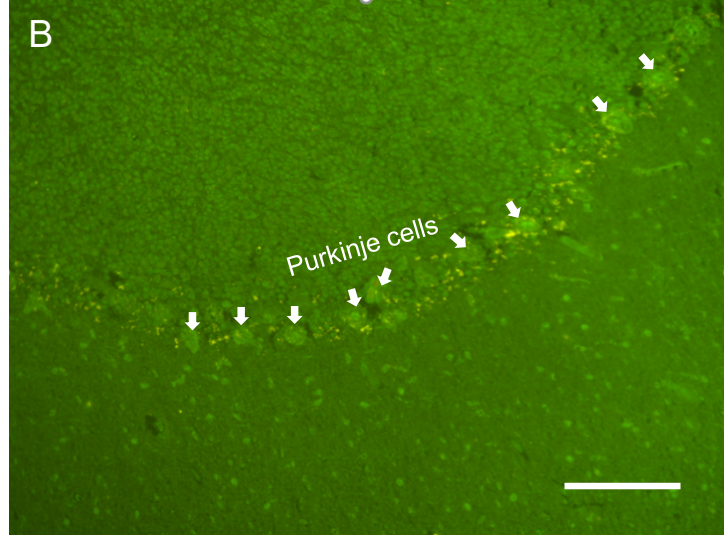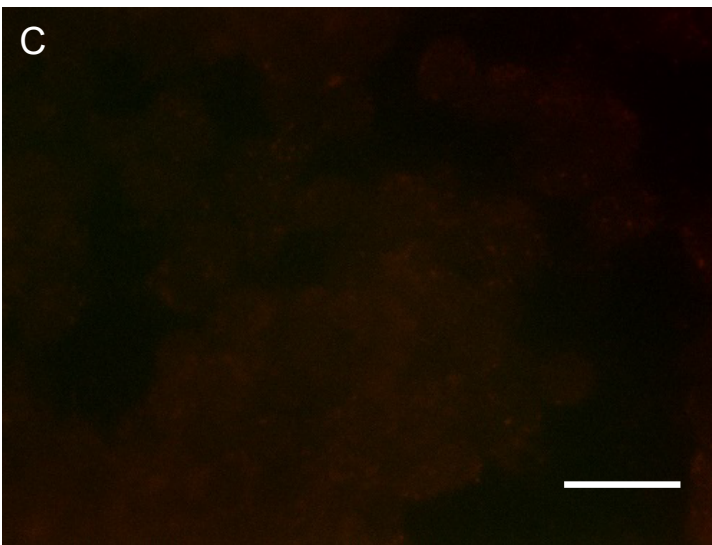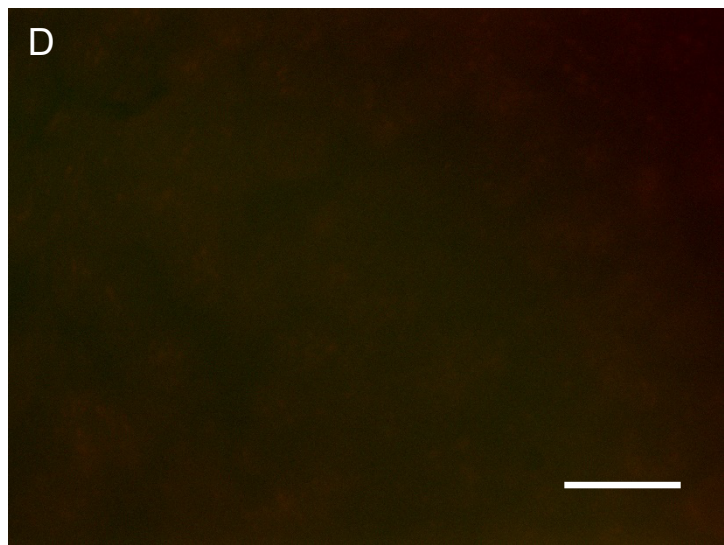

Supplement: Supplementary Figure 1 — Tissue-based indirect immunofluorescence assay (TBA) detected weak fluorescence responses, with specific fluorescence responses in (A) interstitial connective tissue (arrows) from the monkey optic nerve section as well as (B) Purkinje cells (arrows) from the monkey cerebellum brain section (scale bar 100μm). Cerebrospinal fluid MOG (C) and anti-NMDAR (D) antibodies were negative by cell-based assay at 8 months of follow-up (scale bar 100μm). [file Image_1.pdf]

One month

Three months

Eight months

Right

Left

A

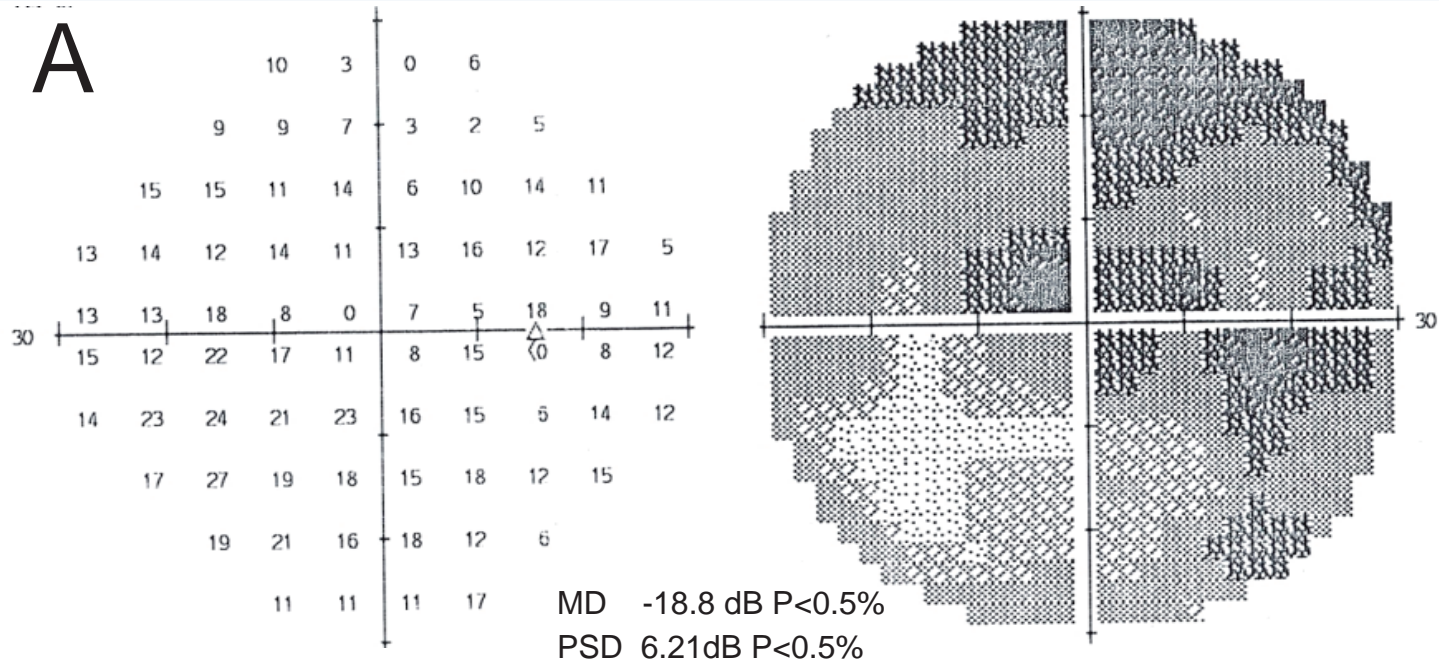

B

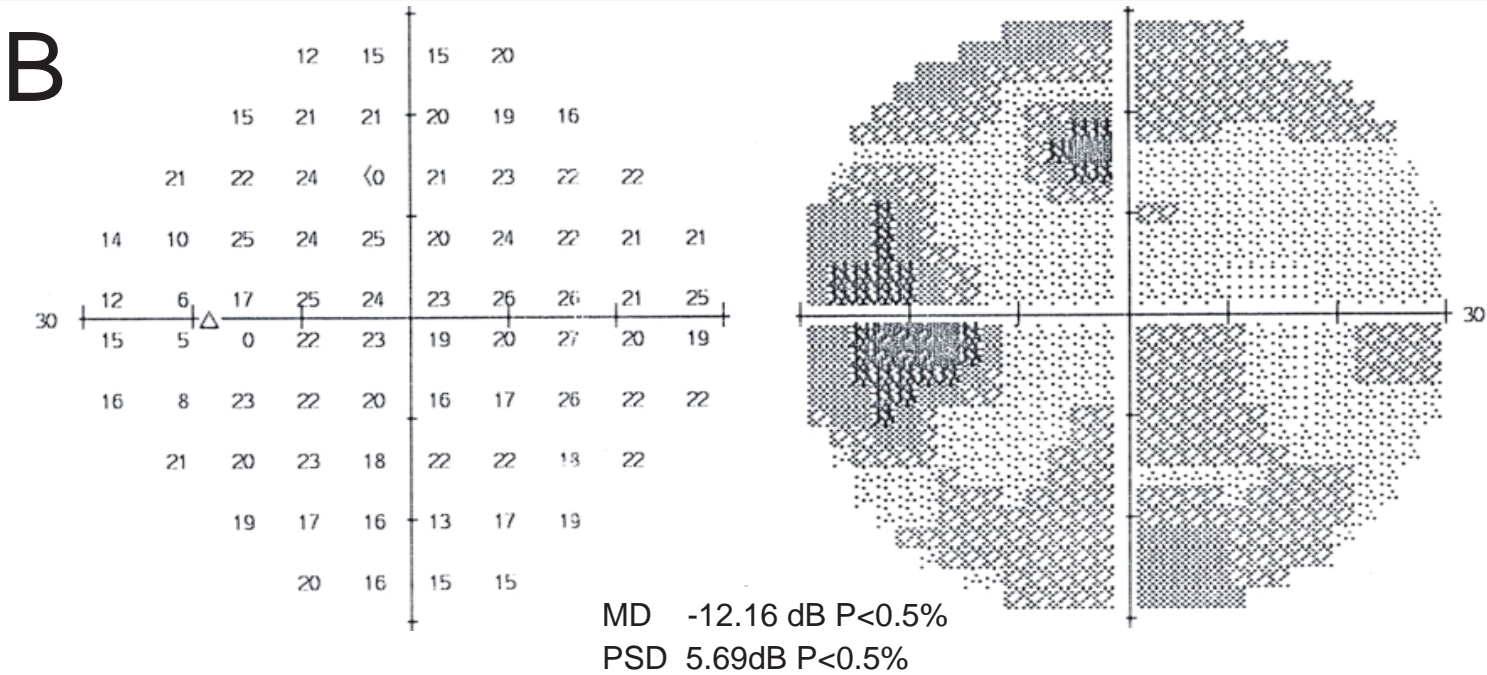

C

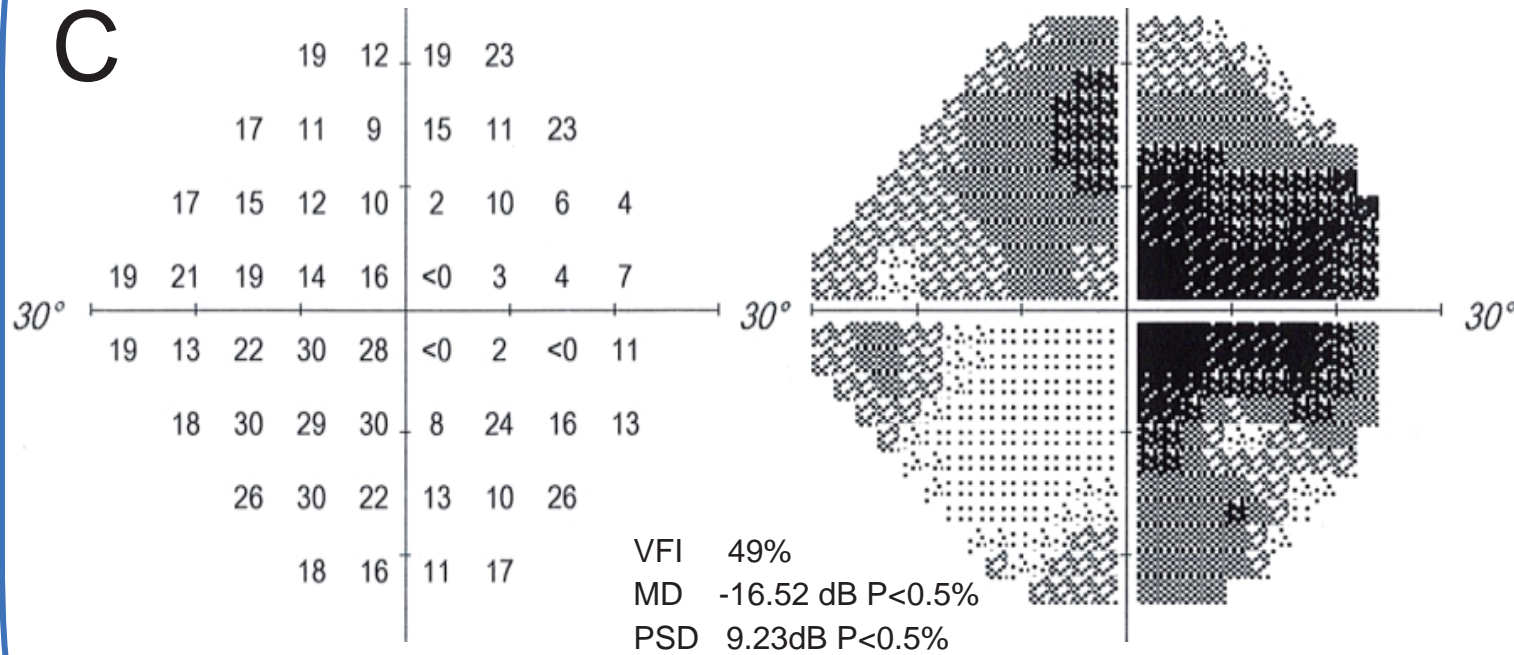

D

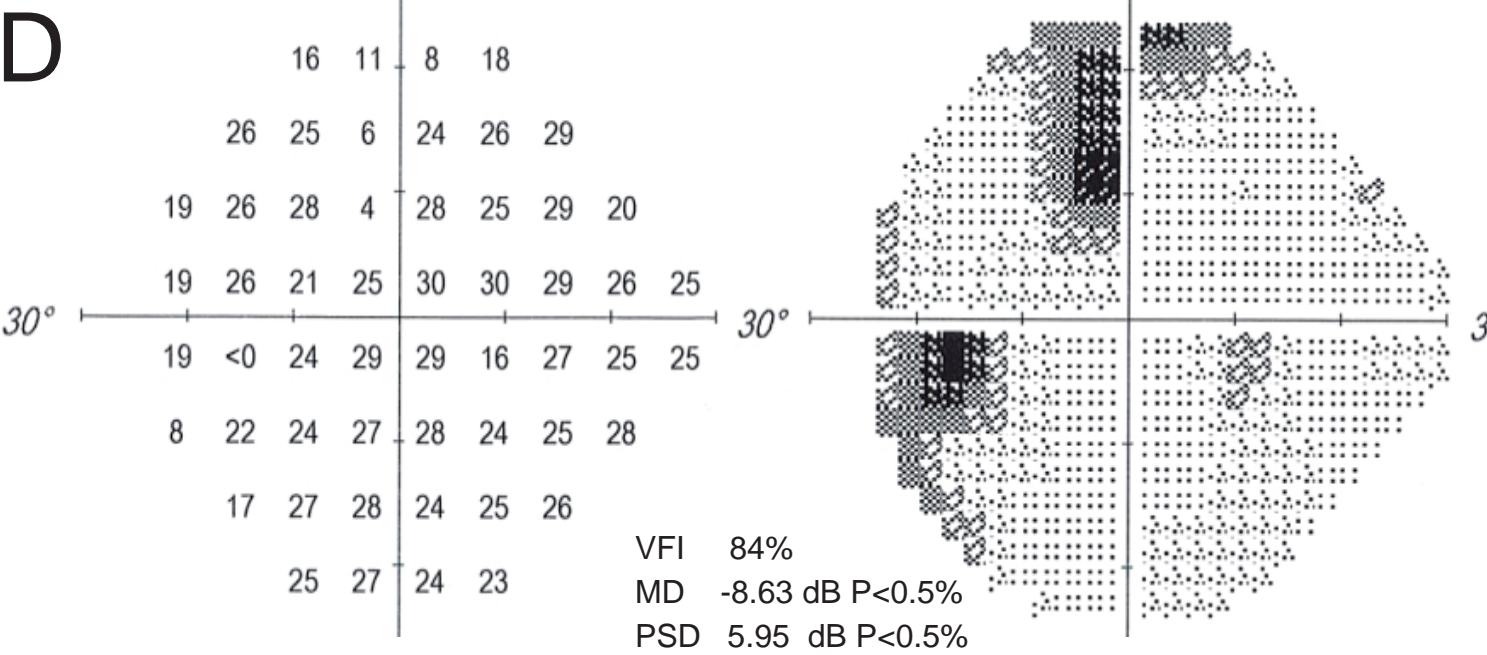

E

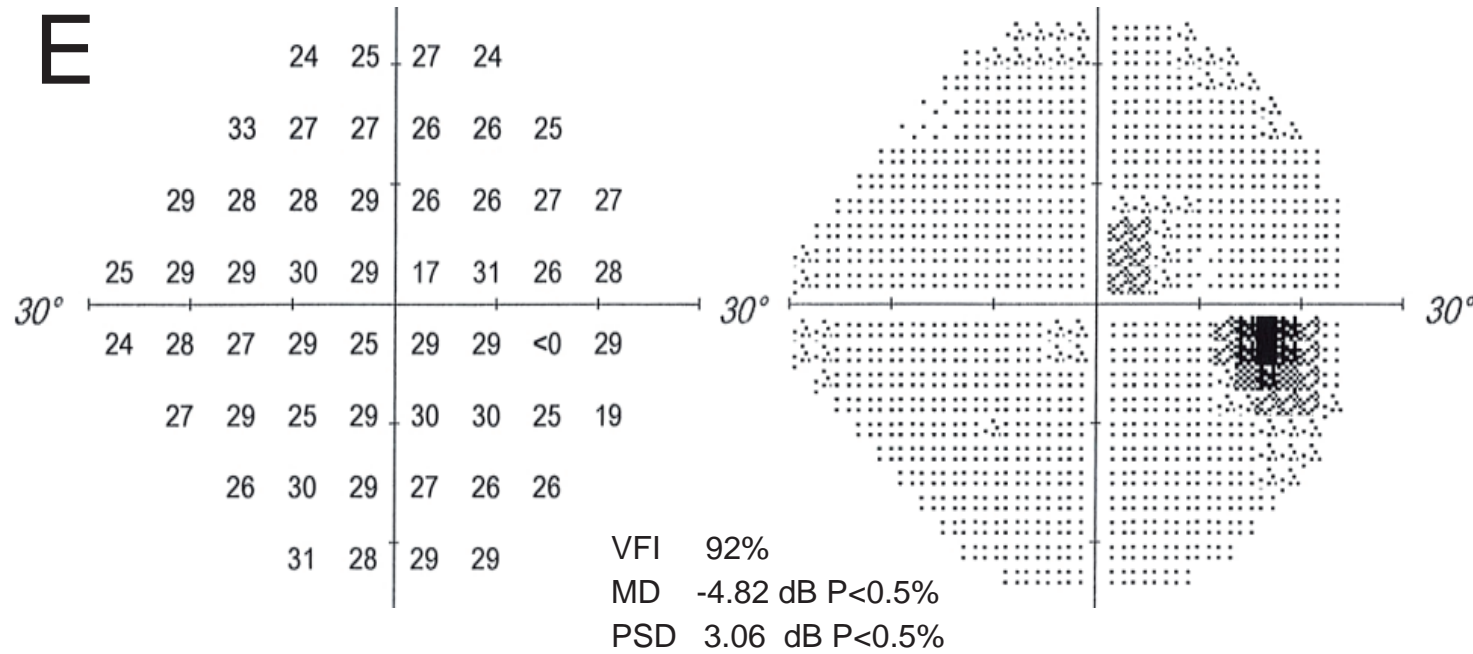

F

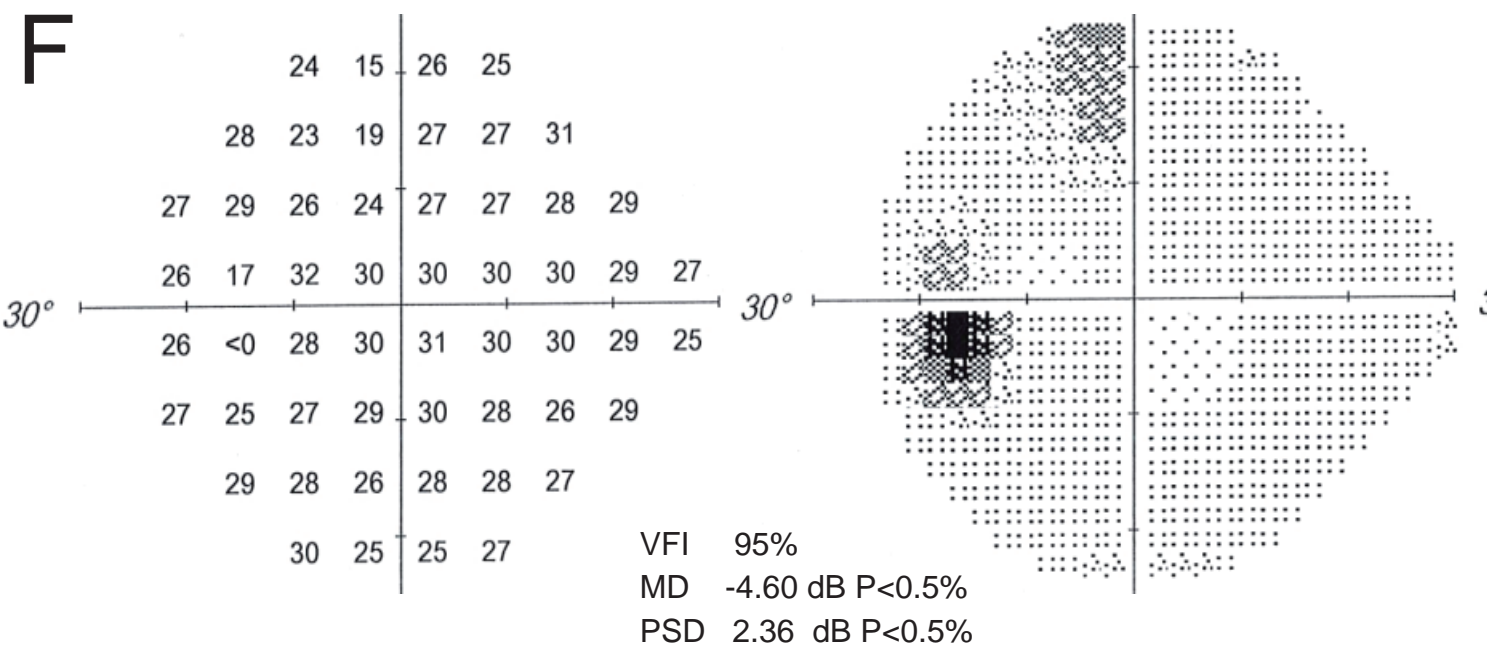

Supplement: Supplementary Figure 2 — Changes of visual filed in different periods after onset: (A, B) one month, (C, D) three months, and (E, F) eight months. [file Image_2.pdf]
