# Supplementary material for: Case Report: Overlapping Syndrome of Anti-NMDAR Encephalitis and MOG Inflammatory Demyelinating Disease in a Patient With Human Herpesviruses 7 Infection
Source: Front Immunol. 2022 Apr 22;13:799454. doi: 10.3389/fimmu.2022.799454 (PMC9074690; doi:10.3389/fimmu.2022.799454)
Supplement: Supplementary file 3 [file DataSheet_1.docx]

**Supplementary file**

**Sample**

Blood and cerebrospinal fluid samples from the patient were collected when he was first admitted to our hospital and during the follow-up period, which were centrifuged at 500g for 15min and stored in -80℃ refrigerator. In addition to the frozen samples used for the later detection of MOG antibody, the rest of the experimental items were fresh samples.

**Next generation sequencing**

Next generation sequencing (NGS) metagenomics were performed at the Center for Precision Medicine, West China Hospital, Sichuan University. Nucleic acid components in the samples were detected to identify pathogenic microbial genome sequences that could exist in the samples, including 6350 kinds of bacteria (including Mycobacterium, Mycoplasma, Chlamydia and Rickettsia), 4945 kinds of viruses, 1064 kinds of fungi and 234 kinds of parasites with known genome sequences. Methods: The meta NGS detection based on Illumina/ SOL-Exa Genome Analyzer platform was used to analyze the microbial nucleic acid sequences in the samples and identify the microorganisms by comparing them with the known microbial nucleic acid sequences in the database. High-throughput sequencing procedures include nucleic acid extraction, RNA enrichment, reverse transcription, two-strand synthesis, library construction, sequencing, bioinformatics analysis, and report interpretation. All the final results were confirmed by RT-PCR.

**Assays for autoantibodies by cell-based assay.**

Detection Kit for Autoimmune Encephalitis Antibody Profile (including anti-NMDARA, anti-LGI1, anti-CASPR2, anti-GABABR, anti-AMPAR, anti-DPPX, anti-mGluR5) by transfected cell-based assay. Commodity item numbers: NI01010-DA (EUROIMMUN, Lübeck, Germany).

Detection Kit for Central Nervous System Autoimmune Demyelinating Diseases Antibody Profile (including anti-MOG, anti-AQP4, anti-GFAP) by transfected cell-based assay. Commodity item numbers: NI02005-DA (EUROIMMUN, Lübeck, Germany)

The detected target protein was transfected into HEK293 cells, and the transfected cells were fixed on the cell plate as an antigen matrix. The diluted sample was incubated with a reaction hole coated with the transfected target protein. If the sample contained anti-target protein antibody, it was specifically bound to the corresponding target protein antigen. After the addition of green fluorescent protein (GFP) labeled anti-human immunoglobulin antibody, the target protein-anti-target protein-luciferase labeled anti-human immunoglobulin antibody complex can be formed, and the specific fluorescence pattern can be observed under a fluorescence microscope.

The detection results were observed under a forward or inverted fluorescence microscope and a monochromatic excitation fluorescence module was used. (Green fluorescence: excitation wavelength: 460-550nm, emission wavelength: 590nm). Cell matrix was observed with a 20× objective lens. Excitation filter: 488nm. Results: Average number of positive fluorescence staining cells per 200x field (positive cells were cells with green fluorescence signal, and fluorescence was located on the surface of cell membrane or cell matrix; During counting, five fields should be selected at different positions on the cell chip to obtain the average number of positive cells). Negative: no specific green fluorescent cells. Suspicious positive: 1-10 green fluorescent cells. Positive: 11-50 green fluorescent cells. Moderate positivity: 51-100 green fluorescent cells. Strong positive: > 101 green fluorescent cells.

**Assays for TBA.**

Tissue-based indirect immunofluorescence assay (TBA) slides with monkey optic nerve sections and brain sections were used for performing TBA. Each slide was incubated with 30μl of sample diluted in phosphate-buffered saline (PBS) (1:100) at 4℃ for 3 h, flushed with PBS, and thereafter immersed in PBS for 5 min. Subsequently, polyclonal goat anti-human IgG (Cat. ZF-0308, ZSBio, China) labeled with fluorescein isothiocyanate (FITC), was incubated at room temperature for 30 min. The slides were then washed again, embedded in glycerol (approximately 10μl per cryosection), and were thereafter examined under a DMi8 microscope (Leica, Germany).
