# Supplementary material for: Case Report: Overlapping Syndrome of Anti-NMDAR Encephalitis and MOG Inflammatory Demyelinating Disease in a Patient With Human Herpesviruses 7 Infection
Source: Front Immunol. 2022 Apr 22;13:799454. doi: 10.3389/fimmu.2022.799454 (PMC9074690; doi:10.3389/fimmu.2022.799454)
Supplement: Supplementary file 4 [file Table_1.docx]

**Supplementary Table 1 Additional auxiliary examination results**

| **Cerebrospinal fluid** | **(CSF)** |
| --- | --- |
| Glucose | **(-)** |
| Chlorine | **(-)** |
| Fungus (smear/culture) | **(-)** |
| Bacteria (smear/culture) | **(-)** |
| Acid-fast bacilli cultures | **(-)** |
| Malignant cell | **(-)** |
| Flow cytometry | **(-)** |
| **^a^ Neuronal cell surface antibodies** | **(CSF/serum)** |
| Anti-NMDAR | **(+/-)** |
| Anti-GABAB | **(-/-)** |
| Anti-LGI1 | **(-/-)** |
| Anti-CASPR2 | **(-/-)** |
| Anti-AMPAR | **(-/-)** |
| Anti-DPPX | **(-/-)** |
| Anti-mGluR5 | **(-/-)** |
| **^a^ Onconeuronal antibodies** | **(CSF/serum)** |
| Anti-Hu | **(-/-)** |
| Anti-Ri | **(-/-)** |
| Anti-Yo | **(-/-)** |
| Anti-GAD | **(-/-)** |
| Anti-PCA2 | **(-/-)** |
| Anti-Ma2/Ta | **(-/-)** |
| Anti-CV2/CRMP5 | **(-/-)** |
| Anti-SOX1 | **(-/-)** |
| Anti-amphiphysin | **(-/-)** |
| **Oligoclonal bands** | **(-/-)** |
| **Anti-AQP4** | **(-/-)** |
| **Anti-MOG** | **(+/-)** |
| **Tumor markers** | **(Serum)** |
| Prostate-specific antigen | **(-)** |
| α-fetoprotein | **(-)** |
| Carcinoembryonic antigen | **(-)** |
| Carbohydrate antigen 125 | **(-)** |
| Carbohydrate antigen 19-9 | **(-)** |
| Carbohydrate antigen 724 | **(-)** |
| **Microbiological exams** | **(Serum)** |
| Human cytomegalovirus | **(-)** |
| Epstein-Barr virus | **(-)** |
| Human immunodeficiency virus | **(-)** |
| Syphilis | **(-)** |
| Mycobacteria | **(-)** |
| **Immunological test results** | **(Serum)** |
| Anti-nuclear antibody | **(-)** |
| Anti-keratin antibodies | **(-)** |
| Anti-dsDNA | **(-)** |
| Anti-Smith antibodies | **(-)** |
| Anti-SS-A and SS-B | **(-)** |
| Anti-ANCA | **(-)** |
| Anti-anticardiolipin antibodies | **(-)** |
| Rheumatoid factor | **(-)** |
| Anti-topoisomerase antibody | **(-)** |
| **C-reactive protein** | **(-)** |
| **Erythrocyte sedimentation rate** | **(-)** |
| **Thyroid function and antibodies** | **(Serum)** |
| Triiodothyronine | **(-)** |
| Thyroxine | **(-)** |
| Free triiodothyronine | **(-)** |
| Free thyroxine | **(-)** |
| Thyroid-stimulating hormone | **(-)** |
| Thyroid globulin antibody | **(-)** |
| Thyroid peroxidase antibody | **(-)** |

NMDAR, N-methyl-D-aspartate; GABAB, Anti-gamma-aminobutyric acid-B receptor; LGI1, leucine-rich glioma-inactivated protein 1; CASPR2, contactin-associated protein-like 2; AMPAR, α-amino-3-hydroxy-5-methyl-4isoxazolepropionic acid receptor; DPPX, Dipeptidyl peptidase-like protein-6; mGluR5, metabotropic glutamate receptor 5. ANCA, anti-neutrophil cytoplasmic antibodies; dsDNA, double-stranded deoxyribonucleic acid; MOG, Myelin oligodendrocyte glycoprotein; AQP4, Anti-aquaporin 4; Anti-SS-A and SS-B, anti SS-A(Ro) and anti-SS-B(La) autoantibodies;
